# The antinociceptive effects of ferulic acid on neuropathic pain: involvement of descending monoaminergic system and opioid receptors

**DOI:** 10.18632/oncotarget.7973

**Published:** 2016-03-07

**Authors:** Ying Xu, Dan Lin, Xuefeng Yu, Xupei Xie, Liqun Wang, Lejing Lian, Ning Fei, Jie Chen, Naping Zhu, Gang Wang, Xianfeng Huang, Jianchun Pan

**Affiliations:** ^1^ Brain Institute, School of Pharmacy, Wenzhou Medical University, Wenzhou, Zhejiang Province, 325021, China; ^2^ Department of Pharmaceutical Sciences, School of Pharmacy and Pharmaceutical Sciences, State University of New York at Buffalo, Buffalo, NY 14214, USA; ^3^ Pharmaceutical Engineering and Life Sciences, Changzhou University, Changzhou, Jiangsu Province, 213000, China; ^4^ Department of Clinical Pharmacy, Hangzhou First People's Hospital, Hangzhou, Zhejiang Province, 310006, China

**Keywords:** ferulic acid, neuropathic pain, analgesic effect, monoamine, opioid receptor

## Abstract

Neuropathic pain can be considered as a form of chronic stress that may share common neuropathological mechanism between pain and stress-related depression and respond to similar treatment. Ferulic acid (FA) is a major active component of angelica sinensis and has been reported to exert antidepressant-like effects; however, it remains unknown whether FA ameliorate chronic constriction injury (CCI)-induced neuropathic pain and the involvement of descending monoaminergic system and opioid receptors. Chronic treatment with FA (20, 40 and 80 mg/kg) ameliorated mechanical allodynia and thermal hyperalgesia in von Frey hair and hot plate tasks, accompanied by increasing spinal noradrenaline (NA) and serotonin (5-HT) levels. Subsequent study suggested that treatment of CCI animals with 40 and 80 mg/kg FA also inhibited spinal MAO-A levels. FA's effects on mechanical allodynia or thermal hyperalgesiawas blocked by 6-hydroxydopamine (6-OHDA) or p-chlorophenylalanine (PCPA) via pharmacological depletion of spinal noradrenaline or serotonin. Moreover, the anti-allodynic action of FA on mechanical stimuli was prevented by pre-treatment with beta2-adrenoceptor antagonist ICI 118,551, or by the delta-opioid receptor antagonist naltrindole. While the anti-hyperalgesia on thermal stimuli induced by FA was blocked by pre-treatment with 5-HT1A receptor antagonist WAY-100635, or with the irreversible mu-opioid receptor antagonist beta-funaltrexamine. These results suggest that the effect of FA on neuropathic pain is potentially mediated via amelioration of the descending monoaminergic system that coupled with spinal beta2- and 5-HT1A receptors and the downstream delta- and mu-opioid receptors differentially.

## INTRODUCTION

Neuropathic pain is defined as pain arising as a direct consequence of a lesion or a disease affecting the somatosensory system [1]. Patients with neuropathic pain have symptoms that are reflected by hypersensitivity to innocuous (allodynia) and noxious stimuli (hyperalgesia) [2]. Currently available treatment, such as opioids and non-steroid anti-inflammatory drugs, can produce serious side effects including drug tolerance and gastric ulcer [3, 4]. Indeed, the neuropathic pain is often resistant to common analgesics, but is sensitive to some antidepressants, such as tricyclic antidepressant amitriptyline, the reversible MAO-A inhibitor moclobemide, and more recent serotonin-noradrenaline reuptake inhibitors venlafaxine and milnacipran [5]. The response of patients to “antidepressant” medications implies that neuropathic pain and depression may share common neuropathological mechanism that contributes to the comorbid presentation and respond to similar treatment.

**Figure 1:**
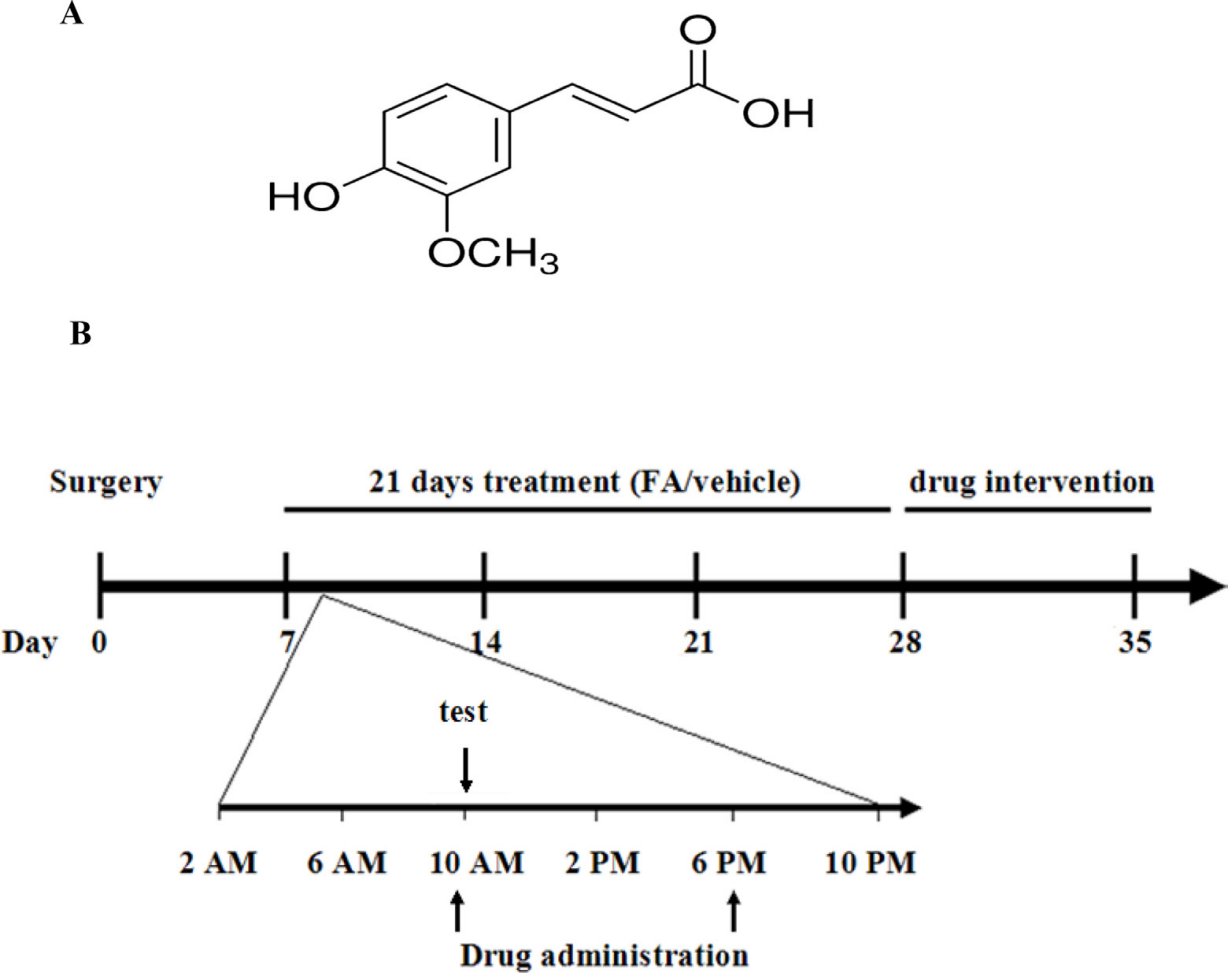
(**A**) Structure of ferulic acid. (**B**) Experimental protocol schedule. On day 7, CCI mice received two oral administrations (10 AM and 6 PM) of ferulic acid (20, 40, 80 mg/kg, p.o.) or vehicle (CMC-Na, p.o.) for 21 days. During treatment, test was done 1 h after the drug administration. After 21 days treatment, antagonists were pre-treated with ferulic acid. Animals were sacrificed immediately after chronic ferulic acid treatment or drug intervention for neurochemical analysis.

The complicated interactions between pain and depression indicate that multi-target therapy may contribute to treatment of neuropathic pain with improved efficacy and better therapeutic profiles. Phytochemicals are such agents that have multiple targets potential for ameliorating pain and the related emotional changes [6]. There are numerous herbal medicines being developed for psychiatric medicines [7–9], such as St. John's wort and curcumin, many of which have comparable efficacy to prescription medications with fewer side effects. Ferulic acid (FA) or 4-hydroxy-3-methoxy-cinnamic acid (Figure 1A) is the majoractive component of extract from Chinese herbal medicine Angelica sinensis. Its pharmacological properties include antioxidant, anti-inflammatory and antidepressant-like activities [10, 11]. Recent study suggested that ferulate inhibits pain and the primary afferent sensitization in the chronic constriction injury (CCI) rats [12]. Our subsequent work supports this finding, which suggests FA increases pain threshold and ameliorates depression-like behaviors through regulating monoaminergic system in the brain [6]. It is possible that pain is alleviated through increasing the activity of the descending inhibitory pathways, such as the spinal monoaminergic and endogenous opioid systems [13–15], which may be compromised in pain condition. However, the underlying mechanism of analgesic effect of FA, i.e. the roles of spinal monoamines, the adrenoceptors and serotonin receptors in regulation of descending inhibitory pathways, remains unknown.

The current study focused our attention upon the descending monoamine system in the spinal cord in elucidating the participation of noradrenergic and serotoninergic receptor subtypes in the analgesic effect of FA. Furthermore, three opioid receptors (delta-, mu- and kappa-opioid receptors) antagonists were used for exploring the possible spinal opioid mechanism in FA's action on neuropathic pain.

## RESULTS

### The effects of chronic FA treatment on mechanical allodynia and thermal hyperalgesia in CCI mice

**Figure 2:**
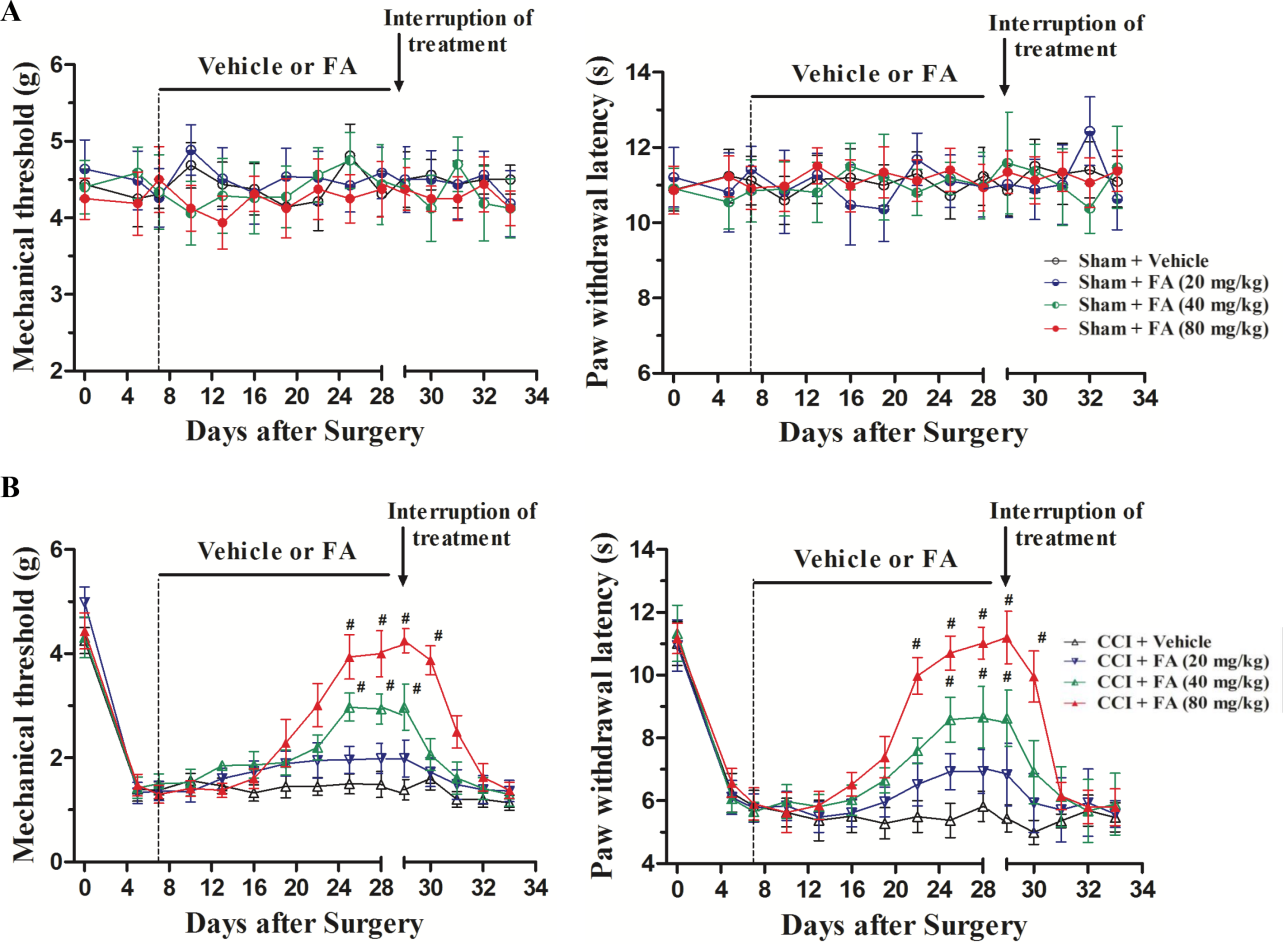
(**A**) The effects of FA treatment (20, 40, 80 mg/kg, p.o., twice a day, 3 weeks) and FA treatment withdrawal on mechanical allodynia and thermal hyperalgesia in sham mice. (**B**) The effects of FA treatment (20, 40, 80 mg/kg, p.o., twice a day, 3 weeks) and FA treatment withdrawal on mechanical allodynia and thermal hyperalgesia in sham mice. Results are expressed as mean ± SEM from 8 mice. ^#^*p* < 0.05 vs. vehicle-treated CCI group.

Figure 2 shows the effects of FA (20, 40 and 80 mg/kg, p.o.) on mechanical allodynia and thermal hyperalgesia in CCI mice. In the sham group, no differences were found in the mechanical thresholds and thermal latency when treatment with different doses of FA (Figure 2A, right and left panels). While in the CCI group, FA (or vehicle) treatment began on day 7 (i.e. 7 days after CCI, Figure 1B), the neuropathic mice exhibited remarkable thermal hyperalgesia and mechanical allodynia. FA increased the mechanical threshold [F_(7, 280)_ = 13.65, *p* < 0.001 for CCI mice; Figure 2B, left panel] and thermal latency [F_(7, 280)_ = 11.86, *p* < 0.01 for CCI mice; Figure 2B, right panel] in time- and dose-dependent manners in CCI mice. The significant effect of FA on mechanical allodynia and thermal hyperalgesia were found 2 weeks after beginning of FA treatment and has lasted for 3 weeks. The maximal effect was achieved when treatment with 80 mg/kg of FA [two-way ANOVA, F_(7, 112)_= 9.69, *p* < 0.001, Figure 2B, left panel] in mechanical threshold; F_(7, 112)_ = 10.44, *p* < 0.01 in thermal latency, Figure 2B, right panel]. Post-hoc analyses showed that FA at 80 mg/kg kept higher threshold both in mechanical allodynia and thermal hyperalgesia between 22–28 days after treatment (*p* < 0.001). However, these effects disappeared when the mice stopped taking the drugs. The pain threshold began to decline 1 day after drug withdrawal, which was back to the same level as CCI surgery mice in 2 days [F_(4, 140)_ = 11.55, *p* < 0.001 for mechanical allodynia; F_(4,140)_ = 13.88, *p* < 0.001 for thermal hyperalgesia; Figure 2B].

### The effects of FA on 5-HT, noradrenaline, dopamine and their metabolites in spinal cord

Monoamines and their metabolites in the spinal cord were measured to evaluate the influence of FA on descending monoamine transmission after three weeks treatment of FA (20, 40 and 80 mg/kg, p.o.). As shown in Table 1, noradrenaline and 5-HT levels were significantly decreased in the spinal cord in CCI mice (*p* < 0.01). FA (20, 40 and 80 mg/kg, p.o.) dose-dependently increased the levels of noradrenaline and 5-HT, compared to that of CCI groups (F_(3,31)_ = 4.402, *p* < 0.05 for 5-HT; F_(3.31)_ = 8.067, *p* < 0.001 for noradrenaline). The noradrenaline and 5-HT levels were increased at the dose of 40 mg/kg (both *p* < 0.05), also increased at the dose of 80 mg/kg (*p* < 0.001 and *p* < 0.05, respectively), without changing the contents of other monoamone/metabolites (MHPG, Dopamine, DOPAC) besides 5-HIAA. 5-HIAA was decreased when CCI-mice were treated with 80 mg/kg FA (*p* < 0.05).

**Table 1:** The effects of ferulic acid (FA) on the concentrations of monoamines and their metabolites in the spinal cord of sham and CCI mice

Group	Dose (mg/kg)	Spinal cord (ng/g tissue)					
		5-HT	5-HIAA	Noradrenaline	MHPG	Dopamine	DOPAC
**Sham**		371.28 ± 46.9	255.83 ± 79	282.41 ± 28.6	226.76 ± 23.9	254.30 ± 49.7	297.18 ± 45.8
**CCI + Vehicle**		188.24 ± 18.0^##^	277.36 ± 26.8	142.25 ± 8.7^##^	223.86 ± 50.7	220.01 ± 34.4	223.86 ± 50.7
**CCI + FA**	20	261.24 ± 43.3	253.53 ± 46.3	202.65 ± 15.7	230.15 ± 31.5	229.06 ± 44.2	248.84 ± 28.6
**CCI + FA**	40	327.70 ± 24.0*	213.36 ± 43.7	235.40 ± 18.7*	256.40 ± 25.8	216.07 ± 37.3	236.24 ± 27.3
**CCI + FA**	80	351.52 ± 45.9**	158.04 ± 38.3*	281.64 ± 32.2***	270.66 ± 50.2	220.12 ± 39.3	256.12 ± 40.5

Table values are expressed as mean ± S.E.M. with units of ng/g for 8 mice in each group.

## *p* < 0.01 vs. vehicle-treated sham mice

* *p* < 0.05

*** *p* < 0.001 vs. vehicle-treated CCI mice.

### The effects of FA on monoamine oxidase activity in sham and CCI mice

Table 2 summarizes the inhibition of type A and type B monoamine oxidase activities by FA in neuropathic mice. After administration of FA at doses of 20, 40 and 80 mg/kg for three weeks, monoamine oxidase-A activity was inhibited in the spinal cord by 13.8%, 29.7% and 37.8%, respectively (F_(3,31)_ = 5.311, *p* < 0.01). The same FA regimen did not affect monoamine oxidase-B activity in CCI mice.

**Table 2:** The effects of ferulic acid (FA) on type A and type B monoamine oxidase activities in the spinal cord of sham and CCI mice

Group	Dose(mg/kg)	Monoamine oxidase-A activity (nmol/30 min/mg protein)	Monoamine oxidase-B activity (nmol/30 min/mg protein)
**Sham + Vehicle**		59.3 ± 3.5	59.8 ± 4.9
**CCI + Vehicle**		92.6 ± 4.8^###^	63.9 ± 3.4
**CCI + FA**	20	79.2 ± 5.4	60.8 ± 5.5
**CCI + FA**	40	68.4 ± 5.2**	61.6 ± 4.6
**CCI + FA**	80	66.3 ± 5.5**	54.4 ± 6.4

Table values are expressed as mean ± S.E.M. with units of ng/g for 8 mice in each group.

### *p* < 0.001 vs. vehicle-treated sham mice.

** *p* < 0.01 vs. vehicle-treated sham mice.

### The effect of descending NA and 5-HT on the analgesic effect of FA in CCI mice

**Figure 3:**
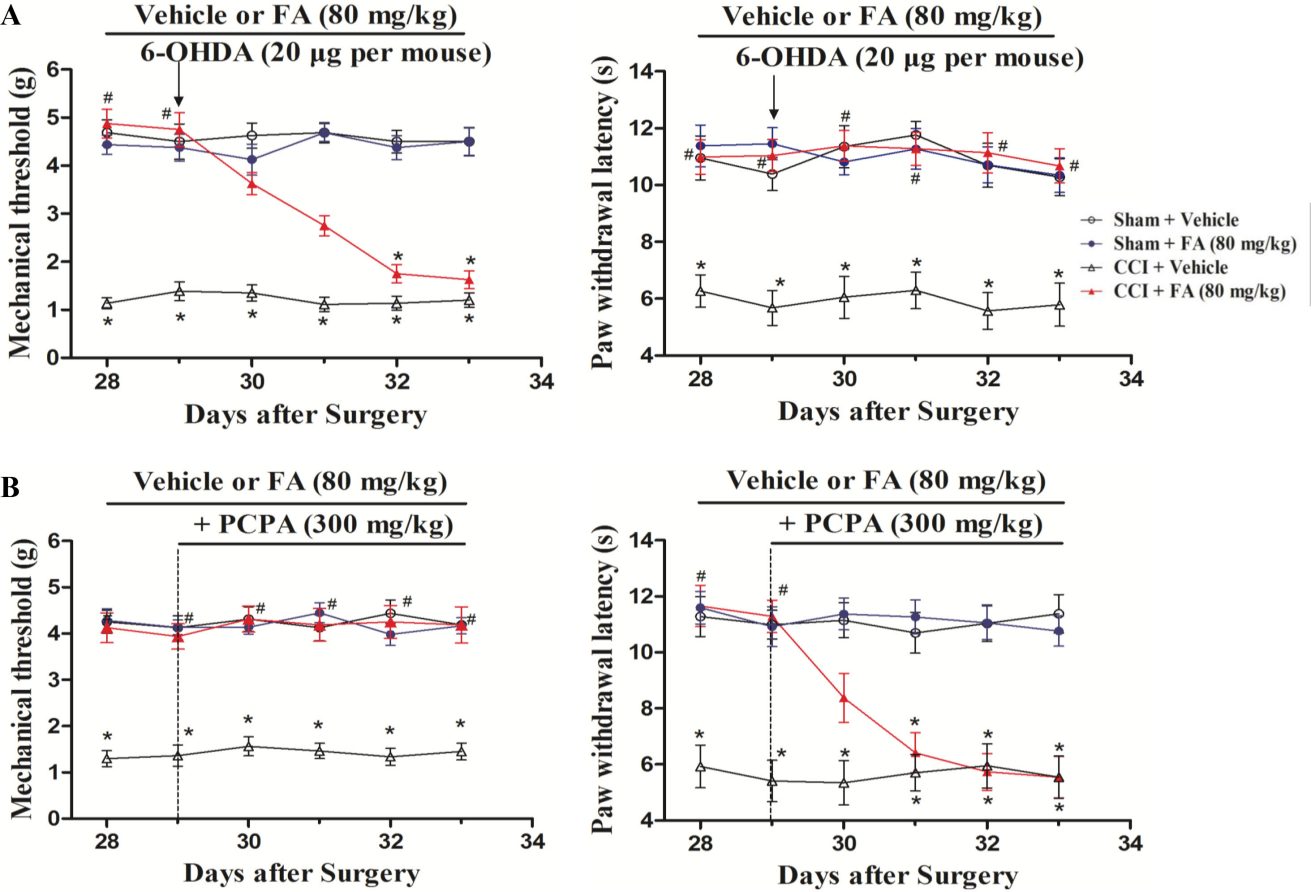
(**A**) The effects of repeated pre-administration of 6-OHDA (20 μg per mouse) on mechanical allodynia and thermal hyeralgesia in sham and CCI mice. (**B**) The effects of repeated pre-administration of PCPA (300 mg/kg) on mechanical allodynia and thermal hyeralgesia in sham and CCI mice. Results are expressed as mean ± SEM from 8 mice. *p < 0.05 vs. vehicle-treated sham group; ^#^*p* < 0.05 vs. vehicle-treated CCI group.

To determine the effect of descending spinal NA and 5-HT on the analgesic action of FA, intrathecal injection of 6-OHDA or intraperitoneal injection of PCPA was used to reduce spinal NA or 5-HT content [16, 17]. Figure 3 shows that injection of 6-OHDA (20 μg, 5 μl per mouse, i.t.), significantly decreased the effects of FA (80 mg/kg) on mechanical allodynia (F_(4, 140)_ = 8.92, *p* < 0.001, Figure 3A, left panel), but did not affect thermal hyperalgesia in the CCI mice (Figure 3A, right panel). Post-hoc analyses show that injection of 6-OHDA utterly abolished the analgesic effect of FA (80 mg/kg) on mechanical allodynia from days 32 to 33 (*p* < 0.01). On the other hand, consecutive injection of PCPA (300 mg/kg, i.p.) completely blocked the analgesic effect of FA on thermal hyperalgesia (F_(4, 140)_ = 8.55, *p* < 0.01, Figure 3B, right panel), without affecting mechanical allodynia in the CCI mice (Figure 3B, left panel). Post-hoc analyses showed that the analgesic effects of FA appeared on thermal hyperalgesia from days 31 to 33 (*p* < 0.01). Notably, 6-OHDA (5 μg and 10 μg per mouse) and PCPA (50 and 150 mg/kg) did not cause any significant effects on mechanical allodynia and thermal hyperalgesia, conforming the doses of PCPA (300 mg/kg) and 6-OHDA (20 μg per mouse) were reasonably (Supplementary Figure 1A and 1B).

### The effect of FA on mechanical allodynia of pre-administration with adrenergic receptors (ARs) antagonists in neuropathic pain

**Figure 4:**
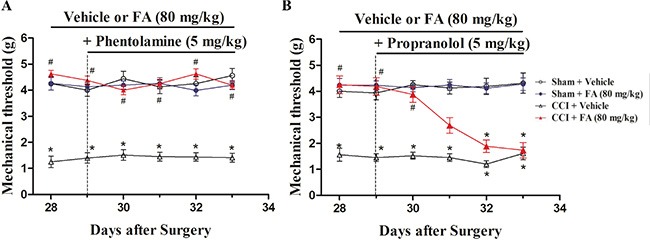
(**A**) The effects of repeated pre-administration of alpha-AR antagonist phentolamine (5 mg/kg) on mechanical threshold in sham and CCI mice. (**B**) The effects of repeated pre-administration of beta-AR antagonist propranolol (5 mg/kg) on mechanical threshold in sham and CCI mice. Results are expressed as mean ± SEM from 8 mice. *p < 0.05 vs. vehicle-treated sham group; ^#^*p* < 0.05 vs. vehicle-treated CCI group.

**Figure 5:**
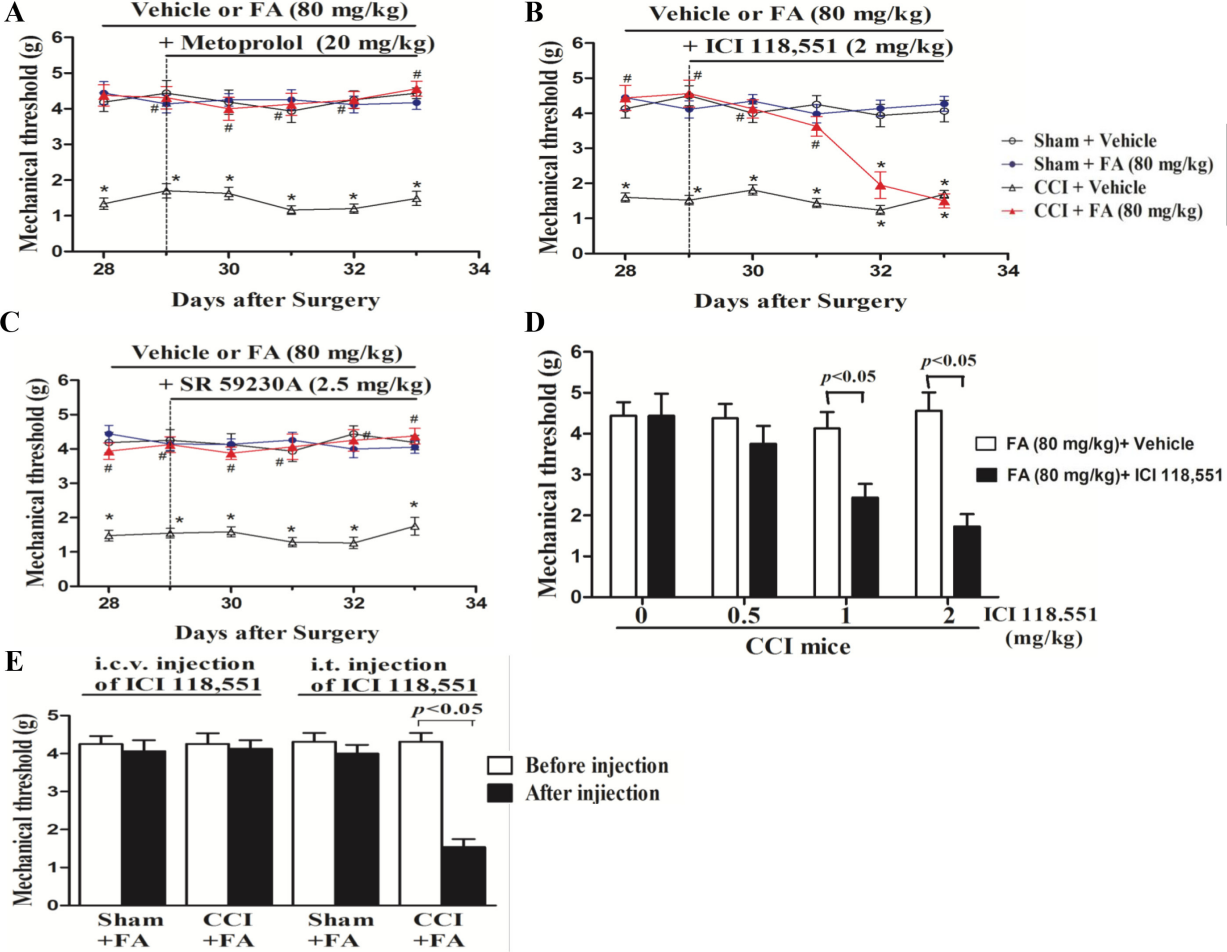
(**A**) The effects of repeated pre-administration of beta1-AR antagonist metoprolol (20 mg/kg) on mechanical allodynia in sham and CCI mice. (**B**) The effects of repeated pre-administration of beta2-AR antagonist ICI118,551 (2 mg/kg) on mechanical allodynia in sham and CCI mice. (**C**) The effects of repeated pre-administration of beta3-AR antagonist SA 59230A (2.5 mg/kg) on mechanical allodynia in sham and CCI mice. (**D**) Effect of pre-administration of different dose beta2 antagonist on mechanical allodynia in FA-treated CCI mice. (**E**) Intrathecal (i.t.), but not itracerebroventricular (i.c.v.) injection of beta2- AR antagonist ICI118551 (0.3 μg/μl for i.t. injection; 5 μg/μl for i.c.v. injection) abolished the analgesic action of FA on mechanical allodynia in CCI mice. Results are expressed as mean ± SEM from 8 mice. *p < 0.05 vs. vehicle-treated sham group; ^#^*p* < 0.05 vs. vehicle-treated CCI group.

To determine which ARs mediate the analgesic effect of FA, different AR antagonists were used to identify whether alpha-ARs or beta-ARs were involved in the analgesic effects of FA. Results show that repeated pre-administration of the nonselective beta-AR antagonist phentolamine (5 mg/kg, i.p.), but not alpha-AR antagonist phentolamine (0.5 and 1.5 mg/kg, i.p.) or beta-AR antagonist propranolol (0.5, 1.5 and 5 mg/kg, i.p.) blocked the effect of FA on mechanical allodynia (F_(6,168)_ = 3.93, *p* < 0.001, Figure 4A and 4B; Supplementary Figure 2), and post-hoc analyses show that injection of propranolol abolished the analgesic effect of FA (80 mg/kg) on mechanical allodynia after pre-treatment for 4 days (*p* < 0.001, Figure 4B), which indicates the beta-ARs, rather than alpha-ARs, mediate the analgesic mechanisms of FA. Therefore, three selective beta-AR antagonists, the beta1-AR antagonist metoprolol (5, 10 and 20 mg/kg, i.p.), beta2-AR antagonist ICI 118,551 (0.5, 1 and 2 mg/kg, i.p.), and the beta3-AR antagonist SR 59230A (0.5, 1 and 2.5 mg/kg, i.p.) were used to ascertain which beta-AR subtypes were responsible for the analgesic action of FA to mechanical stimuli. Results show that the analgesic effect of FA (80 mg/kg) was reversed by pretreatment with the selective beta2-AR antagonist ICI 118,551 (F_(6,168)_ = 7.50, *p* < 0.01, Figure 5B), but not metoprolol or SR 59230A (Figure 5A, 5C; Supplementary Figure 3). Post-hoc analyses show that ICI 118,551 abolished the analgesic effect of FA (80 mg/kg) after pre-treatment for 4 days (*p* < 0.001). Additionally, all antagonists have no effect on mechanical threshold in sham or CCI mice (Figure 5A–5C).

Subsequently, ICI 118,551 was treated by i.t. or i.c.v. to FA-treated CCI mice to locate the functional involvement of the analgesic mechanisms of FA to mechanical stimuli (Figure 5D and 5E). i.t. injection of ICI 118,551 (3 μg in 10 μl), but not i.c.v. injection (10 μg in 2.5 μl) abolished the therapeutic effect of FA (80 mg/kg) on mechanical allodynia (*p* < 0.01, Figure 5E), without influencing on that of sham group.

### The effect of FA on thermal hyperalgesia of pre-administration with 5-HT receptors antagonists in neuropathic pain

**Figure 6:**
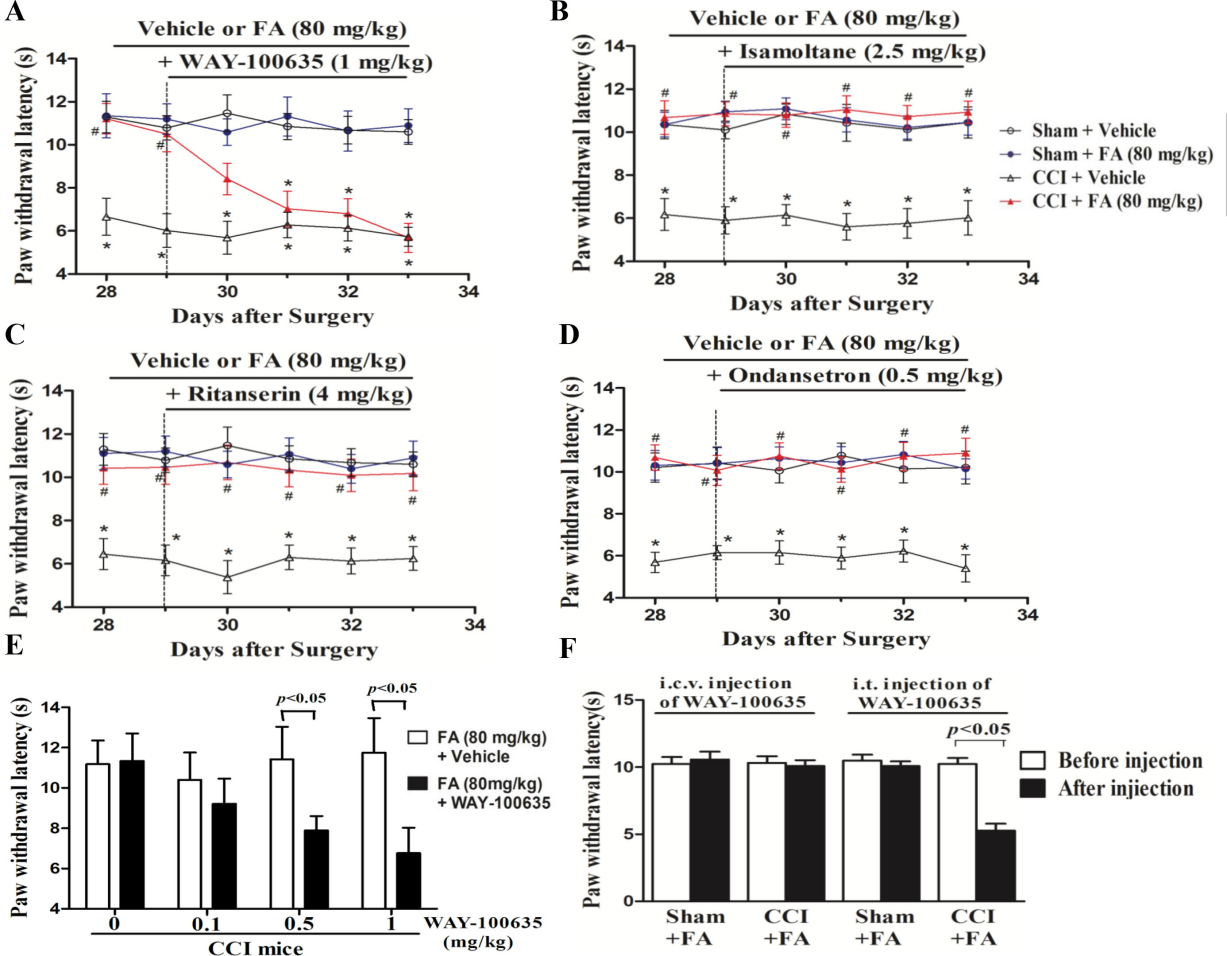
(**A**) The effects of repeated pre-administration of 5-HT_1A_ antagonist WAY-100635 (1 mg/kg) on thermal hyeralgesia in sham and CCI mice. (**B**) The effects of repeated pre-administration of 5-HT_1B_ antagonist isamoltane (2.5 mg/kg) on thermal hyeralgesia in sham and CCI mice. (**C**) The effects of repeated pre-administration of 5-HT_2A/2C_ antagonist ritanserin (4 mg/kg) on thermal hyeralgesia in sham and CCI mice. (**D**) The effects of repeated pre-administration of t 5-HT_3_ antagonist ondansetron (0.5 mg/kg) on thermal hyeralgesia in sham and CCI mice. (**E**) Effect of pre-administration of different dose 5-HT_1A_ antagonist on thermal hyeralgesia in FA-treated CCI mice. (**F**) Intrathecal (i.t.), but not itracerebroventricular (i.c.v.) injection of 5-HT_1A_ antagonist WAY-100635 (0.15 μg/μl for i.t. injection; 2 μg/μl for i.c.v. injection) abolished the analgesic action of FA on thermal hyeralgesia in CCI mice. Results are expressed as mean ± SEM from 8 mice. *p < 0.05 vs. vehicle-treated sham group; ^#^*p* < 0.05 vs. vehicle-treated CCI group.

The monoaminergic system, specifically the one involving 5-HT, plays a multifaceted role in pain modulation, as it has not only produced antinociceptive but also pronociceptive actions [18]. To identify which 5-HT receptors mediate the antinociceptive effect of FA to thermal stimuli, we evaluated the effects of different 5-HT antagonists based on previous studies on antidepressant drugs. We studied the effects of the 5-HT_1A_, 5-HT_1B_, 5-HT_2A/C_ and 5-HT_3_ receptor antagonists on the thermalantinociceptive effect of FA. The analgesic effect of FA (80 mg/kg, i.p.) on thermal hyperalgesia was reduced by repeated pre-administration of the 5-HT_1A_ receptor antagonist WAY-100635 (Figure 6A and 6E; Supplementary Figure 4A). And post-hoc analyses show that WAY-100635 (1 mg/kg) completely abolished the recuperating effects of FA on thermal hyperalgesia in CCI mice after pre-treatment for 4 days (F_(6,168)_ = 7.44, *p* < 0.001). But the anti-hyperalgesic effect of FA was not affected by the selective 5-HT_1B_ receptor antagonist isamoltane (Figure 6B; Supplementary Figure 4B), the selective 5-HT_2A/C_ receptor antagonist ritanserin (Figure 6C; Supplementary Figure 4C), or the selective 5-HT_3_ receptor antagonist ondansetron (Figure 6D; Supplementary Figure 4D).

The further study suggested that WAY-100635 was administered by i.t. or i.c.v. to chronically FA-treated CCI mice, to clarify the regionally discrete roles of the 5-HT_1A_ receptors in the analgesic effects of FA on thermal hyperalgesia. Injection of WAY-100635 via i.t. (1.5 mg in 10 ml) selectively reduced the analgesic effect of FA (80 mg/kg) on thermal nociception in CCI mice (*p* < 0.001), without effects on sham group. However, i.c.v. injection of WAY-100635 (5 mg in 2.5 ml) did not reverse the anti-nociceptive effects of FA on thermal hyperalgesia both in CCI mice and sham group (Figure 6F).

### Acute blockade of delta-opioid receptors abrogated the analgesic effects of FA on mechanical allodynia

**Figure 7:**
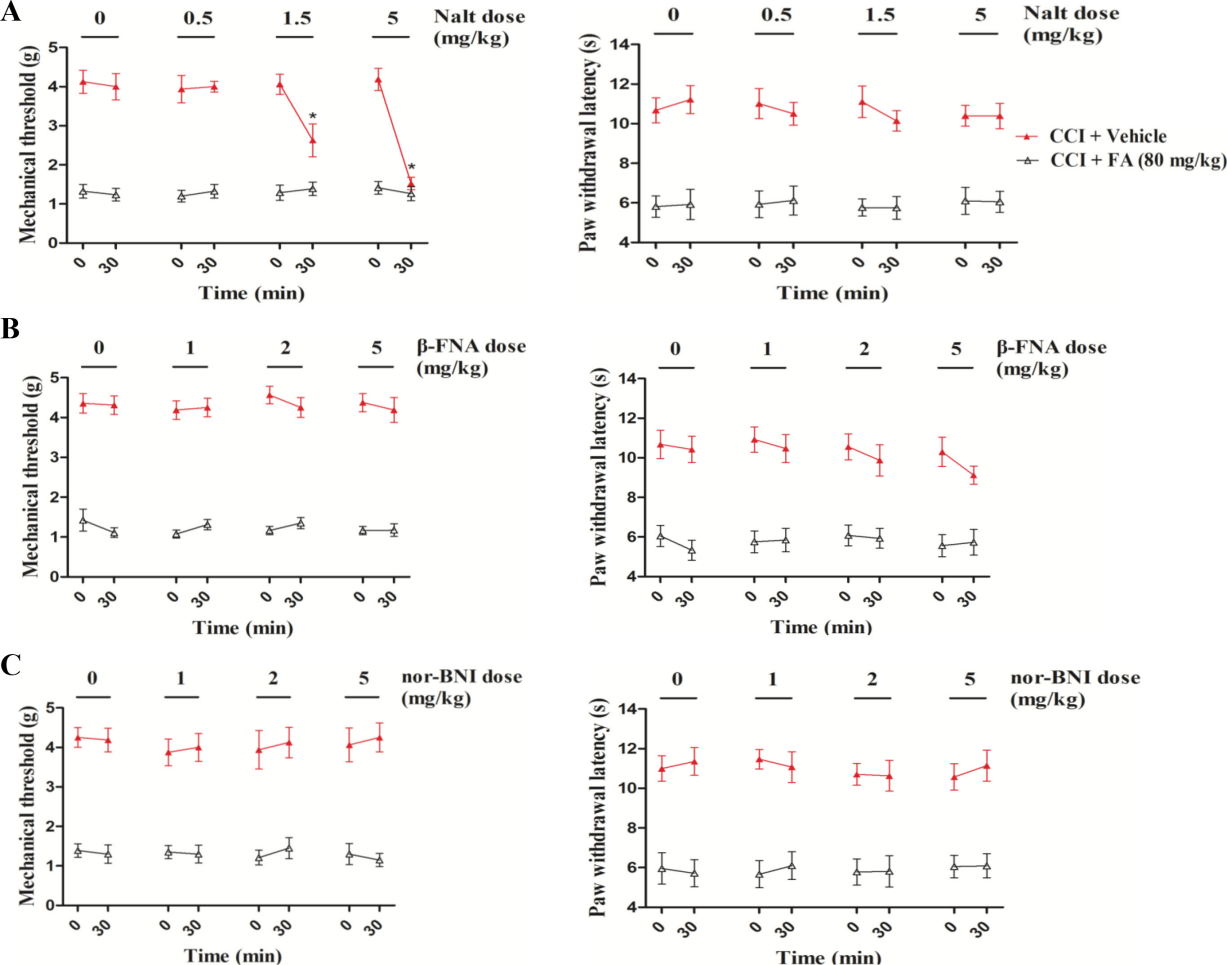
(**A**) The Effects of delta-opioid receptor antagonist naltrindole on mechanical allodynia and thermal hyperalgesia in sham and CCI mice. (**B**) The Effects of mu-opioid receptor antagonist beta-funaltrexamineon mechanical allodynia and thermal hyperalgesia in sham and CCI mice. (**C**) The Effects of kappa opioid receptor antagonist nor-binaltorphimineon mechanical allodynia and thermal hyperalgesia in sham and CCI mice. Results are expressed as mean ± SEM from 8 mice. *p < 0.05 vs. FA-treated CCI group.

To determine whether opioid receptors are involved in the therapeutic effect of FA in neuropathic pain, we used delta-, mu- and kappa-opioid receptor antagonists, with focusing on the discrete contribution to alleviating mechanical allodynia and thermal hyperalgesia. As shown in Figure 7, dose-dependent decreases in the mechanical threshold were observed after administration of the selective delta-opioid receptor antagonist naltrindole hydrochloride (Nalt, 0.5, 1.5 and 5 mg/kg, i.p.) in the FA-treated CCI mice (F_(3,31)_ = 17.18, *p* < 0.001, Figure 7A left), without influencing the therapeutic effect of FA to thermal stimuli (Figure 7A right). In the subsequent experiment, the kappa- and mu- opioid receptor antagonists beta-FNA and nor-binaltorphimine (nor-BNI), at a dosage range of 0.5–5 mg/kg, did not affect the antinociceptive effects of FA (80 mg/kg) on either mechanical allodynia or thermal hyperalgesia in the CCI mice (Figure 7B and 7C, both left and right panels).

## DISCUSSION

Patients suffering from neuropathic pain have a higher incidence of mood disorders such as depression [9, 19]. There are multiple mechanisms participating the etiology and treatment of neuropathic pain and the related mood disorders [20, 21]. The present study explored whether the action of the natural polyphenol FA on CCI-induced neuropathic pain is involved in the activation of descending monoaminergic transmission and the downstream opioid receptors. Chronic FA treatment exhibited modality-specific effect on the CCI-induced chemical allodynia and thermal hyperalgesia via distinct descending monoaminergic mechanisms, i.e. noradrenergic or serotonergic system. Besides its regulation on neurotransmitters evidenced by the neurochemical and biochemical assays, FA was also found to activate the down-regulated beta2-, 5-HT_1A_-, and delta- opioid receptors induced by CCI.

A peripheral nerve injury, such as a nerve section or compression, will result in abnormal sensations and spontaneous pain, which may provoke pain-like allodynia or hyperalgesia. Chronic constriction injury of sciatic nerves was used in the present study, which may allow rapid progress on fundamental aspects of neuropathic pain [22]. Tests for evaluating the neuropathic pain include the von Frey filaments and radiant heat paw-withdrawal tests. The former is valid and useful for measuring the mechanical allodynia by paw withdrawal; the latter appears more valuable when working with unilateral models of pain with manipulations of the sciatic nerve [22, 23]. Present study revealed that FA, a well-known polyphenol, protected sciatic nerves against CCI-induced mechanical allodynia and thermal hyperalgesia ipsilaterally.

The descending monoamine pathway, especially noradrenergic and serotonergic transmission, is a major component of the endogenous pain modulatory system [9, 18]. Chronic constriction of the sciatic nerve results in the decrease in the availability of the neurotransmitters, such as 5-HT and NA, which play critical roles in descending pain modulation pathways [18]. The present study suggested that CCI-induced decreases in spinal 5-HT and NA levels, which were reversed by treatment with FA. This result was consistent with our previous findings that suggested FA exerts analgesic- and antidepressant- like effects through ameliorating monoaminergic function in the reserpine-induced pain-depression mice [6]. The present study revealed that chemical ablation of spinal NA by 6-OHDA abolished the FA's effect on mechanical allodynia; while depletion of 5-HT by consecutive treatment with PCPA for 5 days completely abrogated FA's antihyperalgesic effect against CCI. This modality-specific segregation of FA on nociception was consistent with other studies [17, 24], which indicate the regulation of behaviorally relevant pain modalities by FA might rely on the distinct subsets of descending pain modulation.

The subsequent neurochemical results suggested that the 5-HT turnover, an index of neurotransmitter metabolism, was decreased after high dose of FA treatment (80 mg/kg), indicating a reduction in 5-HT metabolism. A tendency to decrease in NA turnover was also found after treatment with FA. These finding indicates the fact that FA might be exerted its antinociceptive effect through reducing the monoamine decomposition. Indeed, preservation of monoamine neurotransmitters can be achieved either by inhibiting their reuptake or inhibiting their metabolism through the monoamine oxidase (MAO) mechanism [25]. MAO is classified into two types, A and B, according to their sensitivity towards specificity substrates and acetylinic inhibitors. Previous studies suggested that the neurotoxic or CCI induced-hyperalgesia could be attenuated by MAO-A inhibitors [5, 26]. The present study suggested that the increase in spinal MAO-A activity was significant in CCI mice, whereas FA reversed the MAO-A activity dose dependently, which indicates the MAO-A may be closely linked to the dysfunction of descending neurotransmitters underlying neuropathic pain.

In the further drug interaction experiments, we found that the different subtypes of monoamine receptors are responsible for the modality-specificity and the segregating antinociceptive effect of FA. Non-selective beta-AR antagonist propranolol and selective beta2-AR antagonist ICI 118,551, but not the non-selective alpha-AR, beta1- or beta3-AR antagonists abrogated the analgesic effect of FA on mechanical stimuli. These observations were consistent with the previous studies which suggested that beta2-ARs, but not alpha2-, beta1- or beta3-ARs were necessary for various antidepressant drugs to alleviate mechanical allodynia [14, 27]. Interestingly, we found that the blockade of spinal beta2-ARs by intrathecal injection (i.t.), but not supraspinal level by intraventricular injection (i.c.v.) with ICI 118,551, completely abolished the FA's effect on mechanical allodynia. This finding was reinforced by the fact that beta2-ARs are highly expressed in the dorsal horn of the spinal cord, which is a critical relay for pain processing and also provides neuroanatomical substrate for FA. This also implies the importance of the descending noradrenergic tone coupled with spinal beta2-ARs mediated pain inhibitory pathway in the treatment of neuropathic allodynia [28, 29]. The interruption of allodynia usually requires chronic, but not acute, treatment with beta2-AR antagonists [14]. The beta2-AR related delay for therapeutic onset was further confirmed by the present results that suggested the action of FA on allodynia required the chronic treatment (21 days), which was similar to what was observed from some antidepressants against depression and pain disorders [27]. Since activation of beta2-ARs produces antidepressant-like effects, the beta2 neurotransmission appears one of the common pathways by which FA produces both antinociceptive and antidepressant-like properties. This finding indicates that the long-term molecular and neural plasticity may participate in FA-induced antiallodynic processing.

The involvement of 5-HT receptors in hyperalgesic processing remains uncertain. The results from series of drug interaction experiments suggested that treatment with the 5-HT_1A_ receptor antagonist WAY-100635, but not 5-HT_1B_ receptor antagonist isamoltane, 5-HT_2A/2C_ receptor antagonist ritanserin or 5-HT_3_ receptor antagonist ordanstron, eliminated FA-induced antinociception on thermal hyperalgesia. It is also obvious that i.t., but not i.c.v., injection of WAY-100635, selectively blocked the analgesic effect of FA, which emphasizes the participation of descending serotonergic receptors in the FA's action. Indeed, 5-HT-utilizing descending projections are involved in the pathways known to be important for both inhibition and facilitation of nociceptive signals at the spinal level [17]. Inhibition of postsynaptic 5-HT_1A_ receptors could up-regulate autoreceptors in brainstem 5-HT synthesizing neurons, which could reduce the firing of the 5-HT neurons projection to the spinal cord and other brain regions, leading to hypersensibility to nociceptive stimulus [30, 31]. The present results from the drug interaction and neurochemical assays further exemplify the previous studies, which indicate the analgesic effect of FA against CCI might be mediated, at least in part, by stimulation of descending serotonergic transmission, particularly activation of spinal 5-HT_1A_ receptors.

The endogenous opioid system, via the mu-, delta- and kappa-opioid receptors, is important for the analgesic effects of some antidepressants [14, 27, 32–34]. The present results showed that the analgesic action of FA to mechanical stimuli was affected by blockade of delta- opioid receptor but not of mu- and kappa-opioid receptors, which were similar to the observation after sciatic nerve compression [32, 33, 35]. Considering that the previous studies suggested the abrogation of FA's analgesia demands sustained monoamine inhibition [6], it is possible that opioid receptors, particularly delta-opioid receptor, are the downstream target of the monoamine tone. These are supported by the previous studies, which suggested when the descending monoaminergic receptors are activated after treatment with FA, the downstream opioid receptors may also be stimulated [9]. Indeed, the expression of delta- and mu-opioid receptors is segregated in the dorsal root ganglia, which suggests delta-opioid receptors in controlling mechanical pain and mu-opioid receptors in controlling heat pain [18, 36]. However, This dichotomy in mechanical and heat nociceptive controls by opioid receptors is not present when considering the whole organism or a pathological situation [37, 38]. Our results demonstrate an interaction between beta2-ARs and delta-opioid receptors in controlling mechanical pain, which confirm that the opioid receptor as downstream target of the monoamine tone is activated in the mechanism underlying FA's analgesia.

In conclusion, the present results indicate that the analgesic effect of FA is possibly mediated by its modulatory effect on spinal monoamine system (beta2-adrenoceptor and 5-HT_1A_ receptors) and its ability to decrease delta-opioid receptor. Further studies are conducted in our laboratory to elucidate the precise mechanism for FA's action.

## MATERIALS AND METHODS

### Animals

Male ICR mice (20–22 g) were obtained from the Animal Center of Shanghai Branch, Chinese Academy of Sciences. Upon arrival, the mice were group-housed four per cage and acclimatized to a colony room with controlled ambient temperature (22 ± 1°C), humidity (50 ± 10%) and a natural light/dark cycle (12:12 h, lights on 7:00 AM). All experiments were conducted in accordance with the National Institutes of Health Guide for Care and Use of Laboratory Animals (publication No. 85–23, Revised 1985), and approved by the Wenzhou Medical University Committee on Animal Care and Use. Animals were divided into sham group, CCI model group, CCI+FA (dose in mg/kg of 20, 40 and 80, p.o.) group, antagonist + FA-treated CCI group. All animals were not repeatedly used between experiments.

### Chronic constriction injury procedure

The chronic constriction injury and sham procedure were performed based on the previous research with minor modifications [39]. The surgical procedure was performed aseptically under chloral hydrate anesthesia. The nerve was exposed in the mid-region of the hind limb and close to its trifurcation, which was constricted with 3 loose silk thread ligatures until a brief twitch was observed in the ipsilateral hind limb. Sham-operated animals received the same surgical procedure without silk ligature on the nerve.

### Evaluation of thermal hyperalgesia (to heat)

The thermal hyperalgesia was assessed by paw-withdrawal latency to a thermal nociceptive stimulus as described elsewhere [23]. Mice were placed within glass enclosures on a plate, and allowed to acclimate for 30 min before testing. A mobile infrared radiant heat source was placed under the glass plate and focused onto the plantar surface of the affected paw of nerve-injured or sham-operated mice. A digital timer recorded the paw-withdrawal latency. Before CCI surgery, a pretest was performed and those mice with thermal latency below 5 s and over 18 s were rejected.

### Evaluation of mechanical allodynia

The animals were evaluated for mechanical allodynia by a series of von-Frey filaments (0.16, 0.4, 0.6, 0.8, 1, 1.4, 2.5, 3, 4, 6 and 8 g). Mice were placed in a cage over an elevated mesh that gave access to the plantar surface of the paws and adapted to the testing environment for at least 20 min. A positive response was noted if the paw was sharply withdrawn. The test was initiated with the 1 g filament, in the middle of the series. Whenever a positive or negative response to a given filament occurred, the next smaller or higher filament was applied. The mechanical threshold was determined as the minimal force that caused at least three withdrawals observed of five consecutive trials, as described previously [14, 40].

### Drugs and drug administration

Ferulic acid (Figure 1A) was purchased from Sigma-Aldrich. FA was prepared daily by dissolving in sodium carboxyl methyl cellulose (CMC-Na). The mice received two administrations (10:00 AM and 6:00 PM) of FA (dose in mg/kg of 20, 40 and 80, p.o.) or vehicle (CMC-Na) for 3 weeks (Figure 1B). The mice were co-administered with antagonist after 3 weeks of FA or vehicle treatment. The following antagonists (purchased from Sigma-Aldrich) were used: non-selective alpha-AR antagonist phentolamine (0.5, 1.5 and 5 mg/kg, i.p.), non-selective beta-AR antagonist propranolol (0.5, 1.5 and 5 mg/kg, i.p.), beta1-AR antagonist metoprolol (5, 10 and 20 mg/kg, i.p.), beta2-AR antagonist ICI 118,551 (0.5, 1 and 2 mg/kg, i.p.), beta3-AR antagonist SR 59230A (0.5, 1 and 2.5 mg/kg, i.p.), 5-HT_1A_ receptor antagonist WAY-100635 (0.1, 0.5 and 1 mg/kg, i.p.), 5-HT_2A/2C_ receptor antagonist ritanserin (1, 2 and 4 mg/kg, i.p.), 5-HT_1B_ receptor antagonist isamoltane (0.5, 1 and 2.5 mg/kg, i.p.), 5-HT_3_ receptor antagonist ondansetron (0.05, 0.1 and 0.5 mg/kg, i.p.), delta-opioid receptor antagonist naltrindole hydrochloride (0.5, 1.5 and 5 mg/kg, i.p.), mu-opioid receptor antagonist beta-funaltrexamine (1, 2 and 5 mg/kg, i.p.) and kappa-opioid receptor antagonist nor-binaltorphimine (0.5, 2 and 5 mg/kg, i.p.). The doses of these antagonists were based on the previous research [27, 41]. For i.p. injection, these antagonists were dissolved in a volume of 5 ml/kg by 0.9% NaCl. For repeated administered with FA, these antagonists were injected twice per day, 30 min before FA administration. For a single co-administration with FA, the monoaminergic or opioid antagonists were administered 30 min before behavior tests on day 28 [32, 33].

### Intrathecal (i.t.) and intracerebroventricular (i.c.v.) injection

In order to localize the monoamine receptors possibly involved, we did i.t. or i.c.v. with ICI 118,551 or WAY 100635 after 3 weeks treatment with FA. i.t. or i.c.v. was designed on day 21 after FA treatment, which were 30 min before the morning and the evening treatments with FA. All the drugs were dissolved in artificial cerebrospinal fluid (ACSF) with the exception of FA, whose vehicle was ACSF containing 0.5% sodium carboxymethyl cellulose.

The intrathecal injection was performed with disposable 27-gauge needle that was connected to a 50l Hamilton syringe. The site of injection in this experiment was between L5 and L6 near to where the spinal cord ends and the cauda equine begins. The mouse were anesthetized and held firmly by the pelvic girdle in one hand, vertebra was accessed through a hole in the muscle made by the needle. The correct intrathecal localization was confirmed by a sudden flick movement of tail. The total volume of 10 μl was injected intrathecally.

For intracerebroventricular injection, the animals were anesthetized and placed in a stereotaxic frame with flat-skull position. 27 gauge hypodermic needle mated to a 10 μl Hamilton syringe was inserted perpendicularly through the skull into the brain and 2.5 μl of vehicle or drug was injected [42]. The site of injection was 1 mm, from either side of the midline on a line drawn through the anterior base of the ears.

### Depletion of descending noradrenaline (NA) and serotonin (5-HT)

To study the roles of descending monoamine neurotransmitters in the analgesic effects of FA, the mice were injected intrathecally with catecholaminergic neurotoxin 6-OHDA (acute treatment) for chemical denervation of spinal norepinephrine transmission or were injected intraperitoneally with p-chlor-ophenylalanine (PCPA) for denervation of 5-HT after treatment with FA for 3 weeks. 6-OHDA was dissolved in 0.9% ACSF containing ascorbic acid (100 mg/ml) [16, 43]. While PCPA was dissolved in 0.9% physiological saline. For 5-HT depletion, PCPA at a dose of 300 mg/kg was treated (i.p.) once a day for five consecutive days [17].

### Determination of spinal monoamines and metabolites

Mice were decapitated and their spinal cords were rapidly removed after treatment with FA for 3 weeks. The tissue samples were stored at −80°C. The contents of 5-HT, noradrenaline, dopamine and their metabolites were measured by using high-performance liquid chromatography with electro-chemical detection with minor modifications [44]. Each sample was homogenized by ultrasonication in perchloric acid. The homogenate was kept on ice for 1 h and then centrifuged at 12,000 × g for 20 min. The pellet was discarded. An aliquot of 160 μl of supernatant was added to 80 μl of solution (containing 0.2 M potassium citrate, 0.3 M dipotassium hydrogen phosphate and 0.2 M EDTA). The mixture was kept on ice for 1 h and then centrifuged at 12,000 × g for 20 min again. 20 μl of the resultant supernatant was injected into an ESA liquid chromatography system equipped with a reversed-phase C18 column and an electrochemical detector (ESA CoulArray, Chelmstord, MA, USA.). The mobile phase consisted of 125 mM citric acid-sodium citrate (pH 4.3) 0.1 mM EDTA, 1.2 mM sodium octanesulfonate and 16% methanol. The flow rate was 1.0 ml/min. The tissue levels of monoamine were expressed in terms of nanograms per gram of tissue.

### Measurements of monoamine oxidase activity

The monoamine oxidase activity (MAO) was assessed according to our previously established protocol with minormodifications [8]. Briefly, the spinal cords of micewere homogenized with 4 ml of phosphate buffer. The MAO activity in spinal was measured in the presence of either 1 μM deprenyl (type B inhibitor) or clorgyline (type A inhibitor). For lysis of the membranes, 0.2 ml of the tissue homogenate was treated with 0.4 ml Triton X-100, 2.5 ml phosphate buffer. The mixture was incubated at 37°C for 15 min. Then 30 μl of 2.19 mM kynuramine dihydrobromide was added to the mixture as the substrate. The reaction was incubated at 37°C for 30 min and terminated by adding 0.2 ml of 5 M perchloric acid. After cooling and centrifugation at 1500 g for 10 min, an aliquot of 0.5 ml of the supernatant was added to 2.5 ml of 1 M NaOH. The fluorescence intensity was detected with excitation at 315 nm and emission at 380 nm using a fluorescence spectrometer. The concentration of 4-hydroxyquinoline was estimated from a corresponding standard fluorescence curve of 4-hydroxyquinoline. Monoamine oxidase activity was expressed as nmol of 4-hydroxyquinoline formed/30 min/mg protein [45].

### Statistical analysis

All data were presented as mean standard error based on our previous description (The data were subjected to multifactor analysis of variance (ANOVA) or one-way ANOVA. For multifactor ANOVA, the surgery procedure (sham or CCI) and the treatment (saline vs. drug injection) were taken as between-group factors. When needed, the time of measurement (either time course or preinjection vs. postinjection data) was taken as a within-subject factor. The Newman-Keuls test was used for post hoc comparisons. For one-way ANOVA, Newman-Keulstest was used for multiple comparisons to determine whether the means differed significantly between two groups. A value of *p* < 0.05 was considered statistically significant.

## SUPPLEMENTARY MATERIALS FIGURES


