# Supplementary material for: The antinociceptive effects of ferulic acid on neuropathic pain: involvement of descending monoaminergic system and opioid receptors
Source: Oncotarget. 2016 Mar 7;7(15):20455–68. doi: 10.18632/oncotarget.7973 (PMC4991467; doi:10.18632/oncotarget.7973)
Supplement: Supplementary file 1 [file oncotarget-07-20455-s001.pdf]

The antinociceptive effects of ferulic acid on neuropathic pain: involvement of descending monoaminergic system and opioid receptors

Supplementary Materials

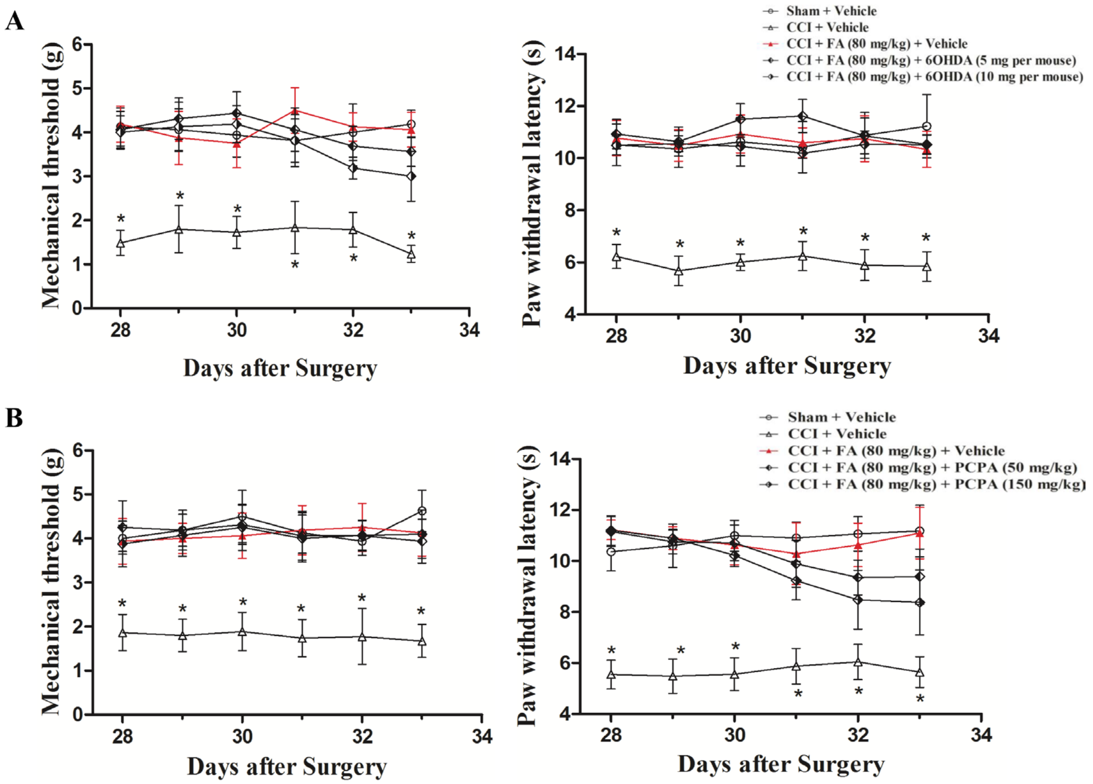

Supplementary Figure S1: (**A**) The effects of repeated pre-administration of 6-OHDA (5 µg and 10 µg per mouse) on mechanical allodynia and thermal hyeralgesia in CCI mice. (**B**) The effects of repeated pre-administration of PCPA (50 and 150 mg/kg) on mechanical allodynia and thermal hyeralgesia in CCI mice. Results are expressed as meanSEM from 8 mice. **p* < 0.05 vs. vehicle-treated sham group.

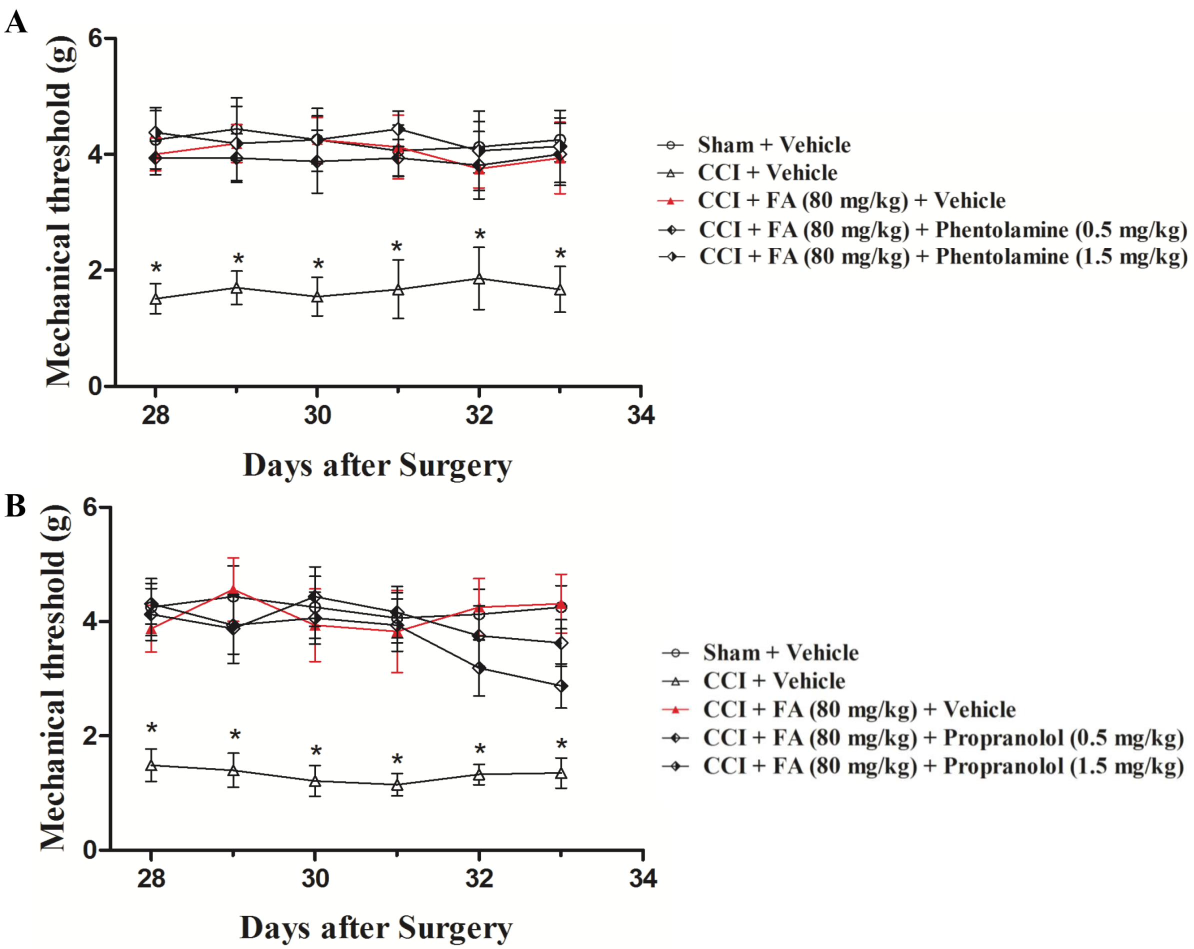

Supplementary Figure S2: (**A**) The effects of repeated pre-administration of alpha-AR antagonist phentolamine (0.5 and 1.5 mg/kg) on mechanical threshold in CCI mice. (**B**) The effects of repeated pre-administration of beta-AR antagonist propranolol (0.5 and 1.5 mg/kg) on mechanical threshold in CCI mice. Results are expressed as meanSEM from 8 mice. *$p < 0.05$ vs. vehicle-treated sham group.

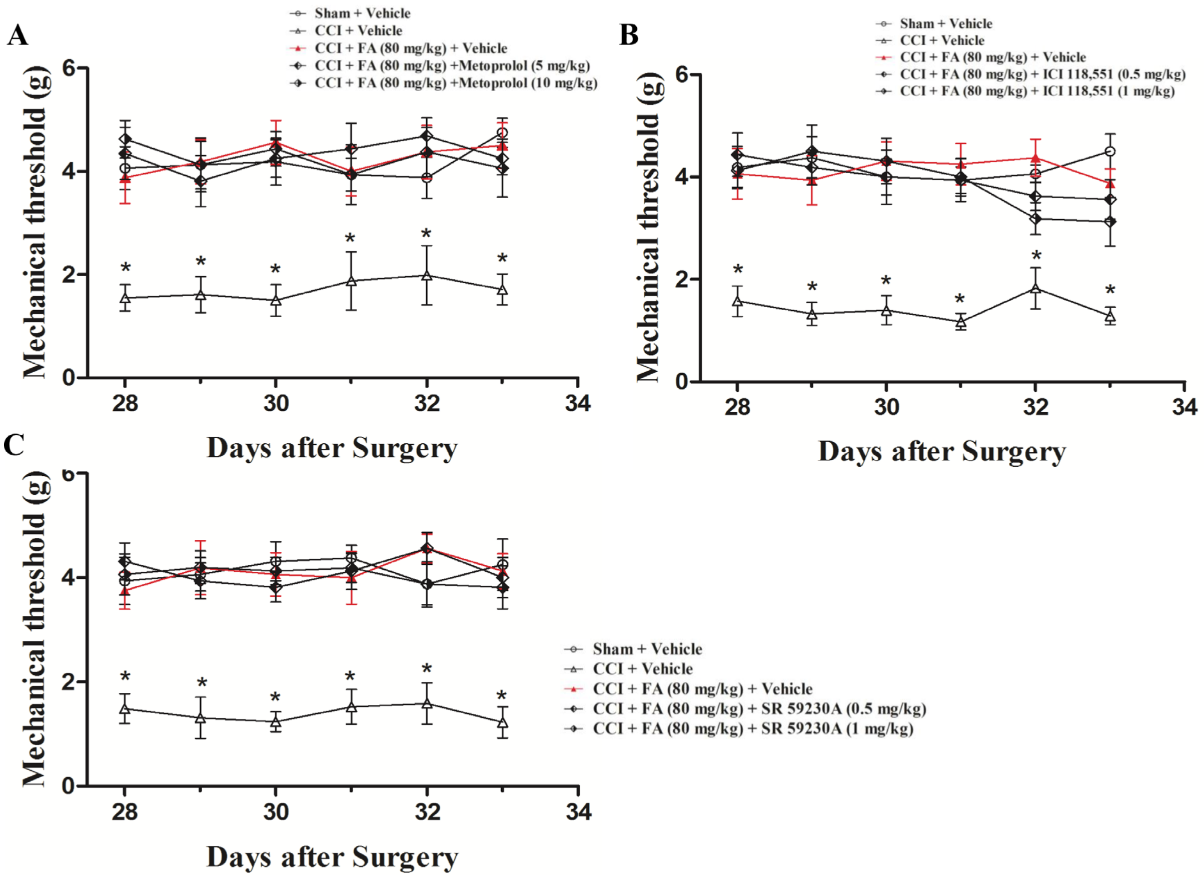

Supplementary Figure S3: (**A**) The effects of repeated pre-administration of beta1-AR antagonist metoprolol (5 and 10 mg/kg) on mechanical allodynia in CCI mice. (**B**) The effects of repeated pre-administration of beta2-AR antagonist ICI118,551 (0.5 and 1 mg/kg) on mechanical allodynia in CCI mice. (**C**) The effects of repeated co-administration of beta3-AR antagonist SA 59230A (0.5 and 1 mg/kg) on mechanical allodynia in CCI mice. Results are expressed as mean ± SEM from 8 mice. *$p < 0.05$ vs. vehicle-treated sham group.

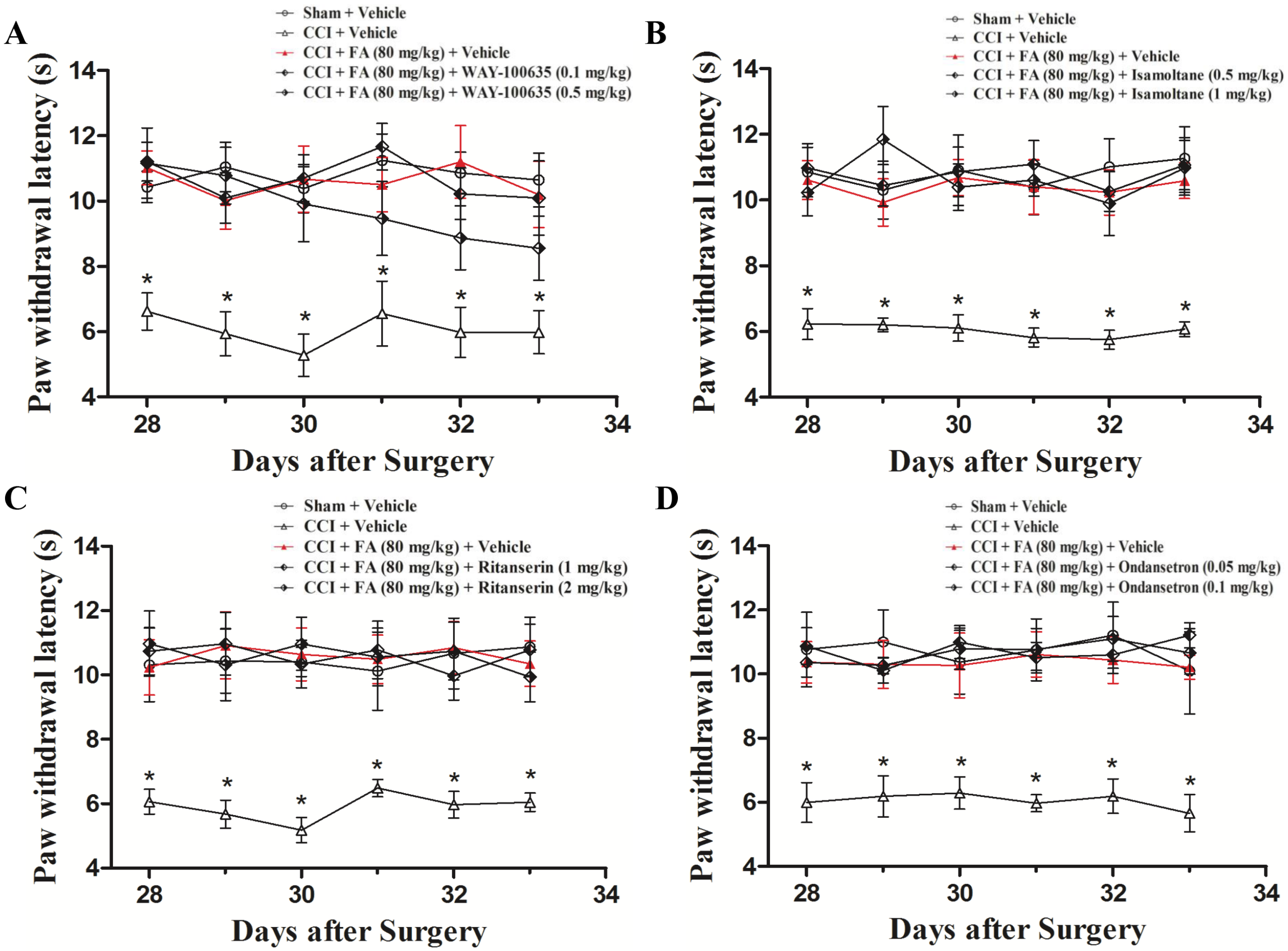

Supplementary Figure S4: (**A**) The effects of repeated pre-administration of 5-HT1A antagonist WAY-100635 (0.1 and 0.5 mg/kg) on thermal hyeralgesia in sham and CCI mice. (**B**) The effects of repeated pre-administration of 5-HT1B antagonist isamoltane (0.5 and 1 mg/kg) on thermal hyeralgesia in CCI mice. (**C**) The effects of repeated pre-administration of 5-HT2A/2C antagonist ritanserin (1 and 2 mg/kg) on thermal hyeralgesia in CCI mice. (**D**) The effects of repeated pre-administration of 5-HT3 antagonist ondansetron (0.05 and 0.1 mg/kg) on thermal hyeralgesia in CCI mice. Results are expressed as mean ± SEM from 8 mice. *$p < 0.05$ vs. vehicle-treated sham group.
